# Congenital adrenal hyperplasia due to P450 oxidoreductase deficiency

**DOI:** 10.3389/fendo.2022.1020880

**Published:** 2022-11-28

**Authors:** Jin Zhang, Kwan Leong Woo, Yongxiong Hai, Shimin Wang, Ying Lin, Ying Huang, Xiaofang Peng, HongShi Wu, Shaoling Zhang, Li Yan, Yan Li

**Affiliations:** ^1^ Department of Endocrinology, Sun Yat-sen Memorial Hospital of Sun Yat-sen University, Guangzhou, China; ^2^ Department of Endocrinology, Jiangmen Central Hospital, Jiangmen, China; ^3^ Department of Endocrinology and Metabolism, Zhuhai People's Hospital, Zhuhai, China; ^4^ Cellular and Molecular Diagnostics Center, Sun Yat-sen Memorial Hospital of Sun Yat-sen University, Guangzhou, China

**Keywords:** congenital adrenal hyperplasia, cytochrome P450 oxidoreductase deficiency, *POR* mutation, c.1379 C>A (p.S460Y) variant, maternal hyperandrogenism

## Abstract

**Objective:**

To raise awareness of Cytochrome P450 Oxidoreductase Deficiency (PORD, a rare form of congenital adrenal hyperplasia (CAH), through a case of pregnant woman with virilization symptoms.

**Case description:**

A 30-year-old Chinese woman was referred to hospital after 7 years of presenting signs of virilization, including voice deepening, acromegaly, hirsutism, clitoromegaly, and acne. These symptoms appeared since her third gestation. Her second birth died 9 hours after birth and had signs of clitoris hypertrophy. Her third born was a son who presented with flat nose, radius and humerus bone malformation, and small penis at birth. Panel of POR-related genetic tests revealed that the patient carried c.1370 G>A (p.R457H), which is a POR heterozygous gene, while her husband carried a POR heterozygous gene as well, c.1379 C>A (p.S460Y). Two heterozygous mutations of the POR were found in her son: c.1370 G>A and c.1379 C>A. In PORD, c.1370 G>A (p.R457H) was reported as a susceptible gene, while c.1379 C>A (p.S460Y) has not been reported as responsible for the disease so far.

**Discussion and literature review:**

PORD is a rare form of CAH and caused by POR gene mutations. Most PORD patients are identified and diagnosed in pediatrics department. Internal medicine and obstetrics physicians are unfamiliar with the disease. As clinical manifestations are diverse, PORD could be easy to miss or to be misdiagnosed. Typical clinical manifestation includes adrenal insufficiency-related symptoms, such as bone malformations and sexual development disorders. PORD is diagnosed through genetic testing. Investigations of steroid metabolic products in urine through gas chromatography-mass spectrometry or liquid chromatography-mass spectrometry are also helpful for the diagnosis, but neither of them are widely available in China. In this case, the patient had a history of infertility, and her third child was born with congenital defect and carried a PORD-related gene. In general clinical practice, if a pregnant woman presents with abnormal virilization symptoms, CAH possibilities should be considered, including rare causes such as PORD.

**Conclusion:**

PORD is a rare autosomal recessive genetic disease. We summarised the clinical characteristics and genotypes that were previously reported in the Chinese population and identified a novel mutation.

## 1 Introduction

Cytochrome P450 oxidoreductase deficiency (PORD) is a relatively rare autosomal recessive genetic disease, which is a rare subtype of congenital adrenal hyperplasia (CAH). Mutations in *POR* affect cytochrome P450 oxidoreductase, an enzyme required for the normal functioning of more than 50 enzymes in the cytochrome P450 family. Thus, *POR* mutations cause a decrease in the activities of various enzymes, leading to disorders of steroid hormone synthesis and a series of clinical symptoms, including abnormal genital development, characteristic skeletal deformities, maternal virilisation during pregnancy, and abnormal secretion of steroid hormones. PORD can be easily missed and misdiagnosed because of the diverse biochemical and clinical manifestations of each subtype of CAH, and because significantly different clinical characteristics result from the different gene mutations. Only approximately 100 cases of PORD have been reported worldwide (1–5), some of which have been temporarily misdiagnosed as CYP17A1 deficiency, CYP19A1 deficiency, or CYP21A2 deficiency. Therefore, in addition to common aetiologies, rare aetiologies should also be considered in the diagnosis of CAH, including PORD.

We here report the case of a mother with virilisation during pregnancy, who gave birth to two children with PORD successively, after delayed diagnosis and treatment. Her son was found to have a previously unreported mutation, c.1379 C>A in *POR* (p.S460Y). Here, we analysed the gene mutation type in the context of their clinical characteristics, and also summarised and analysed the clinical characteristics of 20 Chinese PORD patients previously reported to facilitate a deeper understanding of the disease.In addition, we have identified an unreported mutation locus in the Chinese population, which adds to the genetic diagnosis of PORD in the Chinese population.

## 2 Case description

### 2.1 Case 1 (The mother, carrier of POR heterozygous gene)

The index case was a 30-year-old female who was admitted to the Endocrinology Department of our hospital in May 2020 due to development of a deep voice and enlarged hands and feet that had developed 7 years ago. She signed an informed consent form after admission.

The patient began presenting signs of irilization since the 16^th^ week of her second pregnancy 7 years before admission. She developed voice deepening, facial acne, mandibular prognathism, enlarged hands and feet (with an increase in shoe size from 39 to 42), clitoromegaly and a significant increase in pubic and armpit hair, and other abnormal symptoms. At that time, the patient delivered a full-term baby girl (see Case 2) by caesarean section, who died within hours of birth. After the delivery, the irilization manifestations of the patient significantly diminished compared to earlier, but the deep voice, large hands and feet, and mandibular prognathism were not significantly relieved. No abnormality in sex hormones was found in tests performed 42 days after delivery. The patient had a third pregnancy 2 years before presenting to our institution, and no noticeable signs of irilization emerged during that pregnancy. She delivered a full-term baby boy with PORD (Case 3).

The patient had no significant change in body weight over the past 7 years. She had undergone resection of benign nodules on the left thyroid lobe 12 years earlier. She denied a history of administration of glucocorticoids and had no history of exposure to radioactive substances. Her menstruation cycles were regular and occurred approximately every 30 days, and the age of menarche was 14 years.

She had a reproductive history of gravida 4 (2 miscarriages and 2 full-term births), para 2 (G4P2). One boy is still alive. She had an induced abortion in the first trimester 8 years ago when she had her first pregnancy, because she took medicine by mistake on account of abdominal pain. At term with her second pregnancy 7 years prior to presentation, she gave birth to a baby girl with clitoromegaly, who died within a few hours after birth. Later, 2 years before presentation, with her third pregnancy, she gave birth to a full-term baby boy who was later diagnosed with PORD by genetic testing. She underwent an induced abortion in the first trimester 1 year before presentation, when she was pregnant a fourth time, because this pregnancy occurred near the time of her last childbirth.

Her spouse was in good health. Neither her parents, nor the patient and her spouse, were consanguineous. Other than her son who was diagnosed with PORD, there was no history of similar disease in her family.

#### 2.1.1 Physical examination

At presentation, her blood pressure was 116/82 mmHg and her pulse was 89 beats per minute. Her height was 165 cm, and her weight was 83.7 kg, yielding a body mass index of 30.74 kg/m^2^. Her waist circumference was 101 cm, and hip circumference was 112 cm, with a waist-to-hip ratio of 0.90. She had systemic obesity, and no abnormalities in the heart, lungs, and abdomen. No skin atrophy, acne, and purple abdominal striae were found. She had a deep male voice, but no Adam’s apple or beard. Her hair distribution was normal, but not significantly long, and thick hair was found. There was no noticeable increase in pubic hair or armpit hair. The Tanner stages of pubic hair and breasts were PH4 and B4, respectively. The clitoris was enlarged. The external genitalia showed stage I irilization according to Prader staging. No apparent abnormality was found in other parts of the body.

#### 2.1.2 Laboratory and auxiliary examination

No abnormality was found in the blood cortisol, adrenocorticotropin (ACTH), sex hormones, androgen classification, and thyroid function of the patient. Detailed biochemical examination results see **Table 1**.

**Table 1:** Biochmeical examination results of case 1 and case 3.

Case 1 (The mother)				Case 3 (The child)							
	Results	Reference range	Unit	At age of 5months	Reference range	Unit	Before treatment*	After 1 month of treatment*	After 3 months of treatment*	Reference range	Unit
Blood ACTH (08:00)	46	0-46	pg/mL	**12**.00	0–10.12	pmol/L	340.39	/	**112.9**	118.0-618.0	nmol/L
Serum Cortisol (08:00)	502.3	118.60-618.0	nmol/L	340.39	118.6–618	nmol/L	**12.00**	**26.40**	1.27	0-10.12	pmol/L
Testosterone	0.69	0.35-2.60	nmol/L	**0.96**	0–0.7	nmol/L	/	/	/	/	/
Progesterone	0.44	0.31-1.52	μg/L	/	/	/	/	/	/	/	/
Prolactin	11.96	3.34–26.72	μg/L	10.99	2.1–17.7	ng/ml	/	10.42	/	2.1-17.7	nmol/L
Estradiol	34	19.86–148.13	ng/L	**108.00**	0–73	pmol/L	/	**85.43**	/	0-73.4	pmol/L
Follicle stimulating hormone	8.38	3.85–8.78	IU/L	**3.14**	0.26–3.0	IU/L	/	1.55	/	0.26-3.0	IU/L
Luteinizing hormone	2.19	2.12–10.89	IU/L	**2.64**	0.02–0.3	IU/L	/	**1.12**	/	0.02-0.3	IU/L
Dehydroepiandrosterone sulfate	212.6	98.80–340.0	μg/dl	**0.97**	2.17–15.2	μmol/L	/	/	/	/	/
17-hydroxyprogesterone (17OHP)	1.21	0.1-0.8	ng/ml	**7.2**	< 14.0	nmol/L	/	/	/	/	/
Dihydrotestosterone (DHT)	219.3	24.0–368.0	pg/ml	/	/	/	/	/	/	/	/
Androstenedione	2.55	1.22–8.73	nmol/L	**<1.05**	1.0–11.5	nmol/L	/	/	/	/	/
Anti-Müllerian hormone	1.03	2.80–6.30	ng/ml	/	/	/	/	/	/	/	/
Sex hormone-binding globulin	64.39	32.4–128.0	nmol/L	/	/	/	/	/	/	/	/
24-h urinary 17-ketosteroid excretion	12.9	6–25.0	mg/24 h	/	/	/	/	/	/	/	/
24-h urinary 17-hydroxysteroid excre	tion 2.8	2.0–10.0	mg/24 h	/	/	/	/	/	/	/	/
AST	16	15-40	U/L	/	/	/	/	/	/	/	/
ALT	10	Sep-50	U/L	/	/	/	/	/	/	/	/
Total Protein	**64.2**	65.0-85.0	g/L	/	/	/	/	/	/	/	/
Albumin	**38.3**	40.0-55.0	g/L	/	/	/	/	/	/	/	/
Total bilirubin	5.8	3.4-22.2	μmol/L	/	/	/	/	/	/	/	/
Direct bilirubin	1.3	0.0-3.4		/	/	/	/	/	/	/	/
γ-GGT	11	Oct-60	U/L	/	/	/	/	/	/	/	/
ALP	54	45-125	U/L	/	/	/	/	/	/	/	/
Creatinine	52	44-133	μmol/L	/	/	/	/	/	/	/	/
Uric Acid	300	120-452	μmol/L	/	/	/	/	/	/	/	/
Glucose	5.3	3.9-5.6	mmol/ L	/	/	/	**6.30**	/	**7.6**	4.1-5.9	mmol/L
CO2CP	25	22-31	mmol/ L	/	/	/	/	/	/	/	/
TG	0.82	0.31-2.30	mmol/ L	/	/	/	/	/	/	/	/
CHOL	4.12	2.90-6.00	mmol/ L	/	/	/	/	/	/	/	/
HDL-C	1.4	0.80-1.96	mmol/ L	/	/	/	/	/	/	/	/
LDL-C	2.46	1.30-3.60	mmol/ L	/	/	/	/	/	/	/	/
Blood Sodium	3.84	3.5-5.3	mmol/ L	/	/	/	**136.8**	/	138.0	138.0-144.0	mmol/L
Blood Potassium	141.4	137-147	mmol/ L	/	/	/	4.54	/	3.68	3.4-5.7	mmol/L

Case 2 lacked the relevant data because she died after birth.

*The affected child was administered hydrocortisone acetate in doses from 1.6 mg tid, and the dose was adjusted according to the patient’s condition. During the paediatric follow-up, the levels of blood ACTH and sex hormones returned to normal.

On ultrasound, the uterus was slightly enlarged. The nature of the hypoechoic area (10 mm × 6 mm × 8 mm) in the right ovary and of the cystic hypoechoic area (14 mm × 10 mm × 8 mm) in the left ovary remained to be examined. She had undergone a partial left thyroidectomy, and sonographic images of the thyroid manifested diffuse involvement. A mixed nodule (about 6 mm × 4 mm × 6 mm) was seen in the middle of the right thyroid lobe (ACR-TIRADS 2). Plain and contrasted enhanced computed tomography of the adrenals showed no bilateral adrenal abnormalities. Fiberoptic laryngoscopy indicated chronic pharyngitis and possible laryngopharyngeal reflux.

##### 2.1.2.1 Diagnosis of the patient

Upon genetic analysis, the patient was found to be a heterozygous carrier of mutation in *POR* (1370C>A heterozygote, normal phenotype). She was diagnosed with obesity, a right thyroid nodule, post-partial thyroidectomy (left lobe), a possible ovarian cyst, and chronic pharyngitis.

##### 2.1.2.2 Treatment and follow-up

The patient was advised to lose weight through lifestyle intervention and to undergo genetic counselling with prenatal diagnosis during subsequent pregnancies.

### 2.2 Case 2 (The daughter of Case 1, suspected with PORD)

Case 2 was the daughter of Case 1 and was a child with suspected PORD. This second child was a baby girl born at full term by caesarean section (in 2013). At birth, she was found to have abnormal signs of clitoromegaly, a protruding forehead, and a flat-bridged nose. She died within 9 hours of birth.

### 2.3 Case 3 (The son of Case 1, diagnosed with PORD)

Case 3 was the son of Case 1 and was a child with PORD. This third child was born full-term by caesarean section (May 2018). At birth, he was found to have abnormal signs: a short penis, chordee (urine discharge from the glans penis during urination), a slightly protruding forehead, a flat-bridged nose, and flexion of the upper limbs that could not be straightened. His biochemical examination results see **Table 1**. X-ray images indicated bilateral radiohumeral synostosis, and congenital developmental deformity was considered. Chromosomal examination revealed a 46 XY karyotype.

### 2.4 POR genetic testing

The peripheral blood of Case 1(the mother), her spouse, and Case 3(her son) were collected for DNA analysis. All coding exons and adjacent sequences of *POR* were detected (Guangzhou Jiajian Medical Testing Co., Ltd., Guangzhou, China). The genetic test results are shown in **Table 2**. High-throughput Next Generation Sequencing (NGS) data analysis found that *POR* in Case 3 included a compound heterozygous missense mutation at 2 loci: c.262G>A in exon 4 and c.1609G>A in exon 13. C.262G>A. The former heterozygous missense mutation leads to changes of glycine at position 88 to serine (p.Gly88Ser, G88S), while the latter heterozygous missense mutation results in a change of glycine at position 537 to serine (p.Gly537Ser, G537S). The results from subsequent Sanger sequencing of the corresponding sites in the parents indicated that the nucleotide variation c.262G>A in exon 4 of *POR* of the patient was inherited from the mother, and the nucleotide variation c.1609G>A in exon 13 was inherited from the father. This was consistent with the NGS results. The mother was heterozygous for *POR* c.1370 G>A (p.R457H), and her spouse was heterozygous for *POR* c.1379 C>A (p.S460Y).

**Table 2:** Genetic test results of Case 1 (the mother), Case 3 (her son) and her spouse.

Gene	OMIM number	HG19 position	Transcript	Nucleotide and amino acid variations	Zygote	Class of ACMG^a^ mutation	Inheritance		
**POR**	124015	chr7: 75614497	NM-000941	c.1370G>A (p.R457H)	Heterozygote	Pathogenic	Mother (Heterozygotes)		
**POR**	124015	chr7: 75614506	NM-000941	c.**1379C**>**A** (**p.S460Y**)	Heterozygote	Likely Pathogenic	Father (Heterozygotes)		
**Test Results**									
**Gene**	**Chromosomes** **Location^c^**	**Reference** **serials**	**cDNA level variations**	**Protein level variations**	**Zygote**	**Origin of mutation**	**Genetic** **modality**	**Class of ACMG mutation**	**Associated disease**
*POR*	chr7: 75614497	NM_001395 413.1	c.1361G>A	p.Arg454His	Heterozygote	Mother	AR^b^	Pathogenic	Antley-Bixler Syndrome accompanied by genital abnormalities and steroidogenic disorders
*POR*	chr7: 75614506	NM_001395 413.1	c.1370C>A	p.Ser457Try	Heterozygote	Father	AR	Likely Pathogenic	

Remarks: a:ACMG:American College of Medical Genetics and Genomics . b:AR:Autosomal recessive.

c:Chromosomes Location:Human reference genome GRCH37/hg19.

NGS data revealed that the patient carries a compound heterozygous variant of POR gene on chromosome 17, one of which is NM_001395413.1: c.1361G>A: p.Arg454His, heterozygous and inherited from the mother. This indicates that the variation from G to A at nucleotide 1361 of the gene coding region results in a variation of protein translation from arginine to histidine at amino acid position 454. And this variant is not included in the 1000 genomes, gnomAD and other databases.

The c.1370 G>A (p.R457H) variant has been reported as a pathogenic variant in multiple PORD-related clinical cases, while the c.1379 C>A (p.S460Y) variant has not previously been reported in clinical cases. This variant is “likely pathogenic” according to the American College of Medical Genetics and Genomics (ACMG) variant classification guidelines (PMID: 25741868) (6).

**Figure 1:**
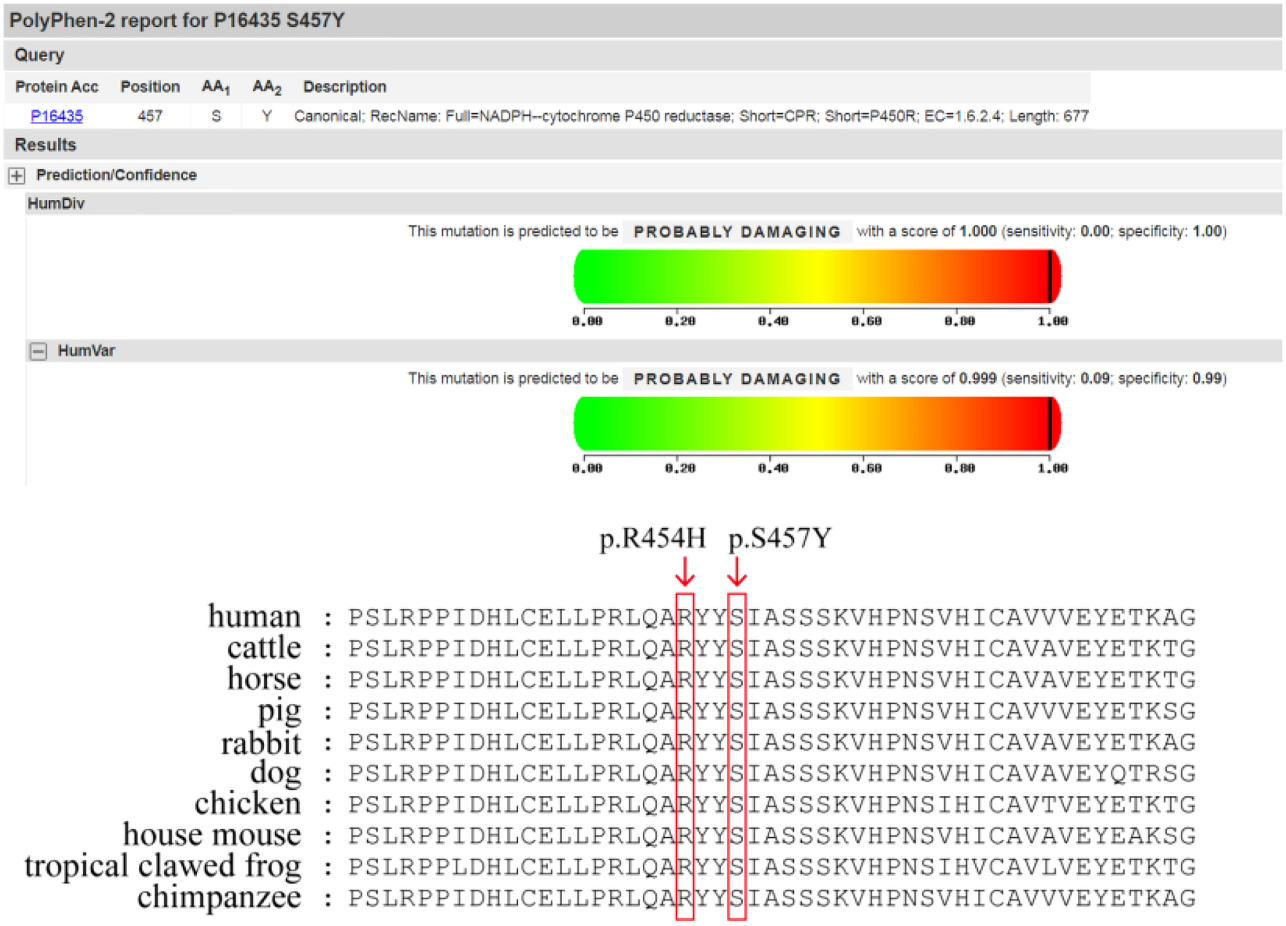
Polyphen2 prediction report & Prediction of protein conservation.

REVEL, Polyphen2, MutationTaster, and other computer prediction software predicted a deleterious variant (REVEL score: 0.959) (**Figure 1**).Meanwhile, the variant has been included in the ClinVar database as a pathogenic/probably pathogenic variant (Variation ID: 16907). The variant has also been identified in Antley-Bixler Syndrome patients (OMIM# 201750) with genital abnormalities and steroidogenesis disorders and has been reported in several publications (7–10) (PMID: 16470797, 20101697, 15793702, 15483095, etc.). Notably, this variant is considered pathogenic according to the ACMG guidelines (2015 edition). Another variant is NM_001395413.1: c.1370C>A: p.Ser454Try, which encompasses a variation in the gene coding region of the nucleotide at position 1370 from C to A, resulting in a change in protein translation from serine to tyrosine at amino acid position 457.

#### 2.4.1 Treatment and follow-up

The affected child was administered hydrocortisone acetate in doses from 1.6 mg tid, and the dose was adjusted according to the patient’s condition. During the paediatric follow-up, the levels of blood ACTH and sex hormones returned to normal.

## 3 Discussion

CAH is a group of autosomal recessive genetic diseases caused by defective enzyme functions due to mutations in genes encoding essential enzymes for steroid hormone synthesis, which eventually results in steroid hormone synthesis disorders. The most common cause of CAH is 21-hydroxylase deficiency (21-OHD) (accounting for more than 90% of the causes), followed by 11β-hydroxylase deficiency (11β-OHD) (accounting for 5–8%). 17-Alpha hydroxylase or 17, 20-lyase deficiency and 3 beta-hydroxydehydrogenase deficiency are less common, accounting for approximately 1% each. Other types of CAH, including PORD, are even rarer. Since the first report in 1985, only about 100 PORD patients have been reported worldwide (1, 11).

Cytochrome P450 oxidoreductase (POR) is a flavoprotein that acts as an electron transporter in the synthesis of various steroid hormones and participates in many physiological reactions of the body (1). Mutations in *POR* can cause impaired activity of the POR enzyme and decreased activities of steroidogenic enzymes in the P450 enzyme system (cytochrome P450 monooxygenases [CYP], including CYP17A1, CYP21A2, and CYP19A1 [aromatase] and other enzymes), eventually giving rise to abnormal secretion of sex hormones and glucocorticoids. Typical clinical manifestations are hermaphroditism at birth, characteristic skeletal developmental malformations, maternal virilisation, and abnormal steroid secretion, without deficiency in mineralocorticoids. However, the clinical manifestations are diverse, and the disease is easy to miss or be misdiagnosed, because the degree of impairment of POR enzyme activity is directly related to the clinical phenotype.

Case 1 (The mother of the PORD foetus) in this study was a phenotypically normal PORD gene carrier with a history of adverse pregnancy, who gave birth to two children (one living) with PORD. The patient was asymptomatic before pregnancy and was only experiencing virilisation in the second trimester (pregnant with a female foetus), and symptoms of virilisation were relieved after delivery.

Pathophysiology process are different during pregnancy of a female foetus and a male foetus and this results in overproduction of androgen and lack of androgen respectively. During pregnancy with a female PORD foetus (with a 46 XX kartoytypoe in general), the reason of virilisation was considered to be caused by excessive androgen elevation through “backdoor bathway”. The underlying mechanisms are mentioned below.

Under normal physiological conditions, POR is involved in the synthesis of cortisol and sex hormones. It catalyses the conversion of progesterone to deoxycortisol and 17-hydroxyprogesterone to 11-deoxycorticosterone in the zona reticularis and zona fasciculata of the adrenal cortex, respectively.

**Figure 2:**
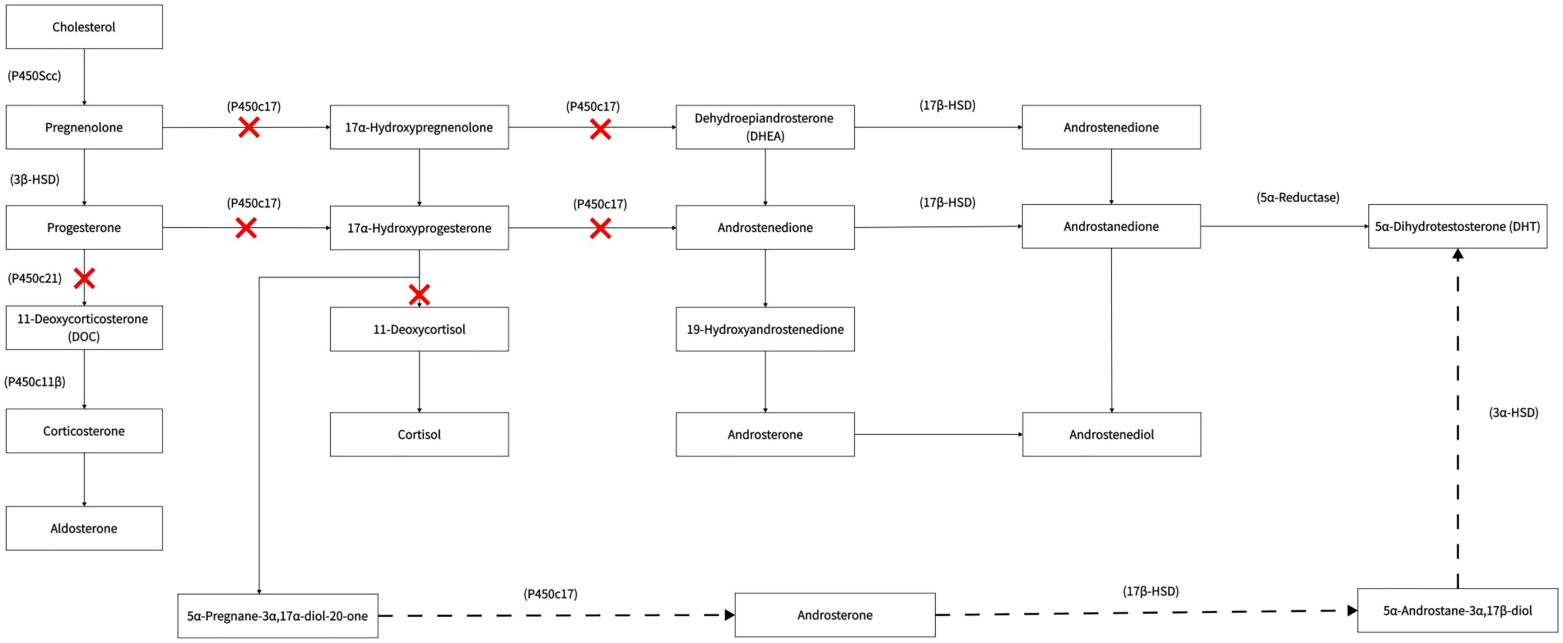
Backdoor pathway.

During pregnancy with a female PORD foetus (with a 46 XX karyotype in general), there is an overproduction of androgen. The conversion of androgen precursors to oestrogens in the body is abnormal due to the decreased activity of various enzymes, such as CYP17A1, CYP21A2, and CYP19A1 (aromatase) in the female foetus; thus, the levels of 17-alpha hydroxyprogesterone synthesised by the foetal adrenals are significantly increased. A large accumulation of 17-alpha-hydroxyprogesterone activates an alternative pathway for DHT synthesis, which can be converted to 5α-pregnan-3α, 17α-diol-20-one, and androsterone *via* this pathway, ultimately resulting in an overproduction of DHT. This pathway does not form part of the conventional androgen production pathway and is known as the “backdoor pathway” (**Figure 2**). The highly active DHT enters the mother’s body *via* the placenta, which can lead to significant virilisation of the mother during pregnancy, possibly accompanied by the excretion of androgen metabolites. The “backdoor pathway” of DHT synthesis is closed as soon as the foetus is born (12). Thus, virilisation during pregnancy can gradually be diminished after childbirth. In addition, our patient’s second, female child also had PORD, and the death within a few hours of birth was presumably attributed to a life-threatening adrenal crisis in the child. This affected baby girl was born virilised. Clitoromegaly occurs in children with 46 XX under the action of high concentrations of DHT. It has been reported that mildly affected female patients have isolated clitoromegaly, while, in severe cases, the clitoris resembles the scrotum (12).

In contrast, during pregnancy with a male foetus(with a 46 XY karyotype in general), there is a lack of androgens synthesised during the embryonic period. In male foetus, POR deficiency affects the activity of 17, 20-lyase, resulting in a significant decrease in the synthesis of androgens, such as the downstream hormones dehydroepiandrosterone, androstenedione, and testosterone. The quantity of androgens received by the mother during pregnancy was reduced accordingly and explained why the mother is not presenting symptoms of virilisation during pregnancy. Due to the lack of male hormones, the baby boy was born with a small penis, congenital chordee, and other reduced masculinity signs. Those with mild symptoms may present with blurred external genitalia and pseudohermaphroditism, and those with severe symptoms may have severe hypospadias or female genitalia.

Furthermore, patients may also have characteristic craniofacial deformities and synostosis in addition to hermaphroditism. The two affected babies in this study had skeletal deformities, such as a slightly protruding forehead and a flat-bridged nose at birth, and the male child also had characteristic deformities, such as synostosis. It has been reported in the literature that about 90% of patients with POR deficiency may have different degrees of craniofacial deformity or specific manifestations, such as synostosis of the long bones of the extremities. Other manifestations include midface hypoplasia, characterised by low-set ears and a pear-shaped nose, craniosynostosis, arachnodactyly, crooked fingers and toes, radiohumeral synostosis, and other synostoses (also known as Antley–Bixler syndrome [ABS]). More severely affected children present with femoral bowing, neonatal fractures, and choanal atresia (4). It is currently believed that skeletal deformities are related to the impaired activity of cholesterol synthesis enzymes in chondrocytes (2). Both lanosterol 14-α-demethylase (CYP51A1) and squalene monooxidase (SQMG) are essential enzymes involved in cholesterol synthesis in bones (11), and defects in their functions lead to impaired cholesterol synthesis in chondrocytes, which can cause defective cell differentiation and increased apoptosis. This is the main cause of skeletal deformities.

The typical clinical manifestations of PORD are ambiguous sex and characteristic skeletal deformities. Male neonates exhibit hypomasculinisation, while female infants exhibit excessive virilisation. Second, patients may present with craniofacial deformities and synostoses, resembling ABS. Moreover, symptoms such as maternal virilisation during pregnancy and sex hormone synthesis disorder in adulthood, which leads to delayed puberty, are also common.

In terms of diagnosis, gas chromatography-mass spectrometry or liquid chromatography-mass spectrometry detection of residual enzyme activity caused by POR deficiency is currently regarded as the gold standard for PORD diagnosis (13, 14). Typical biochemical changes are increased steroid metabolites, such as pregnenolone and progesterone, and 17-OHP metabolites and decreased steroid metabolites, including androgen metabolites. This technique has high specificity and sensitivity and is helpful for the diagnosis and identification of other types of CAH. It allows early detection of increased excretion of pregnenolone and progesterone metabolites in some heterozygous parents. However, at present, only a few laboratories are set up to conduct screening; therefore, screening is challenging (13, 15). The alternative is the chemiluminescence technique, which is frequently used in clinics to detect cortisol and sex steroids but there are limitations on accuracy (15). It is important to note that the hallmark steroids 17OHD and 21OHD may be present in different combinations, which may result in misdiagnosis.

In terms of molecular genetics, genetic testing is helpful for diagnosis and differential diagnosis, particularly in patients with atypical clinical manifestations or in those who cannot be diagnosed by biochemical tests. PORD is an autosomal recessive disorder, and most patients have compound heterozygous mutations in *POR*. The genetic polymorphisms in *POR* have significant racial and individual differences. About 200 *POR* mutations and single nucleotide polymorphisms (SNPs) have been reported to date. Among the types of missense mutations, A287P is the most common *POR* mutation in Caucasians, R457H is highly prevalent in Japanese population (16), while A503V is also common in *POR*, with a prevalence of about 27% in the general population (17).

We also reviewed the clinical data of 20 previously reported Chinese patients with PORD (17–26) (12 female patients) (**Table 3**). After exclusion of patients with missing data, we found that 19 of 19 included patients had abnormal secretion of steroid hormones, 18/19 patients had external genital deformities, 8/19 patients had skeletal deformities, and 10/16 patients had maternal virilisation. Among the 12 pubertal patients, 6/12 cases had delayed puberty, 8/12 cases had delayed growth, and 8/12 cases had ovarian cysts. Understanding of these clinical characteristics can improve awareness of PORD as well as the etiological differentiation of CAH. From the above summary, it can be seen that PORD patients mainly present with varying degrees of abnormal steroid hormone secretion and external genital malformations at birth. And they may habe growth and developmental delays in adulthood, which could be misdiagnosed as other subtypes of CAH. PORD should be considered if patients also have skeletal malformations or virilisation symptoms during pregnancy.

**Table 3:** Summary of gene results and clinical characteristics of 20 Chinese patients with PORD.

Case	Karyotype	POR mutation		Exon	Zygote	Inheritance	Clinical features					Bone deformity	Pubertal failure
		Nucleotide changes	Amino acid changes				Steroidgenesis disorders	Genital abnormalties	Delay of puberty	Maternal virilization	Polycystic ovary /infertility	
**Case 1**	46, XX	c.1370G>A	p.R457H	11	compound	–	Yes	Yes	Yes	Yes	Yes	Yes	No
		c.1493G>C	p.R498P	11	heterozygotes	–							
**Case2**	46, XX	c.1370G>A	p.R457H	11	heterozygotes	Father	–	–	–	Yes	–	–	–
**Case3**	46, XX	–	p.R457H	–	–	–	Yes	Yes	Yes	–	Yes	Yes	Yes
**Case4**	46, XX	c.1370G>A	–	–	heterozygotes	Father	Yes	Yes	–	–	–	Yes	–
		c.917T>G	–	–	–	–							
**Case5**	46, XY	c.262G>A	p.G88S	2	heterozygotes	Mother	Yes	Yes	Yes	Yes	–	Yes	Yes
		c.1370G >A	p.R457H	11	heterozygotes	Father							
**Case6**	46, XX	c.1370G>A	p.R457H	11	homozygotes	Father(heterozygous) Mother(heterozygous)	Yes	Yes	Yes	No	Yes	No	No
**Case7**	46, XX	c.744C>G	p.Y248T	8	heterozygotes	Mother (heterozygous)	Yes	Yes	Yes	Yes	Yes	Yes	Yes
		c.1370G>A	p.R457H	12	heterozygotes	Father (heterozygous)							
**Case8**	46, XX	c.1370G>A	p.R457H	11	homozygotes	–	Yes	Yes	No	No	Yes	No	Yes
**Case9**	46, XX	c.667C >T	p.R223X	–	heterozygotes	–	Yes	No	No	–	Yes	No	No
		c.1820A>G	p.Y607C	–	heterozygotes	–							
Case10	46, XX	c.1370G>A	p.R457H	12	homozygotes	Father (heterozygous) Mother (heterozygous)	Yes	Yes	No	Yes	–	Yes	No
Case11	46, XY	c.1820A>G	p.Y607C	14	heterozygotes	Father(heterozygous)	Yes	Yes	No	No	–	No	No
		c.957-958delTG	–	9	heterozygotes	Mother(heterozygous)							
Case12	46, XY	c.919G>T	–	–	heterozygotes	Mother(heterozygous)	Yes	Yes	–	No	No	No	–
		c.1615G>A	–	–	heterozygotes	Father(heterozygous)							
Case13	46, XX	c.1370G>A	p.R457H	–	–	–	Yes	–	Yes	Yes	Yes	Yes	Yes
		c.744C>G	p.Y248X										
Case14	46, XY	c.1370G>A	p.R457H	–	–	–	Yes	Yes	–	Yes	–	Yes	Yes
		c.744C>G	p.Y248X										
Case15	46, XY	c.1370G>A	p.R457H	–	–	–	Yes	Yes	–	Yes	–	Yes	Yes
		c.1660C>T	p.R554X										
Case16	46, XY	c.1370G>A	p.R457H	–	–	–	Yes	Yes	–	No	–	No	–
		c.1820A>G	p.Y607C										
Case17	46, XY	c.1370G>A	p.R457H	–	–	–	Yes	Yes	–	No	–	Yes	–
		c.629A>G	p.D210G										
Case18	46, XX	c.1370G>A	p.R457H	–	–	–	Yes	Yes	Yes	Yes	Yes	Yes	–
		c.517-19_517-	10delGGCCC TGTGinsC										
Case19	46, XY	c.1370G>A	p.R457H	–	–	–	Yes	Yes	–	Yes	–	Yes	–
		c.517-19_517-	10delGGCCC TGTGinsC										
Case20	46, XX	c.1370G>A	p.R457H	–	–	–	Yes	Yes	–	–	–	Yes	–
		c.1370G>A	p.R457H										

* The (-) symbol indicates the absence of Data.

Case 1 (2008, Peking union medical college hospital), case 2 (patient's mother, 2013, Peking union medical college hospital), case 3 (2014, Peking union medical college) ,case 4 (2016, children's hospital affiliated to Shanghai Jiao tong university), case 5 (2017, Peking union medical college), case 6 (2017, the first affiliated hospital of China medical university), Case 7 (2018, the first affiliated hospital of air force military medical university), case 8 (2018, Ruijin hospital), case 9 (2018, Ruijin hospital), case 10 (2019, children's hospital of Chongqing medical university), case 11 (2019 ,children's hospital of Chongqing medical university), case 12 (2018, Medical Genetics Research Center, School of Life Sciences, South University), Case 12-20 (Beijing Children's Hospital, Capital Medical University in 2019) (the first 7 cases were from Beijing Children's Hospital, and the last case was from Children's Hospital of Zhengzhou Medical University).

Among the 20 reported PORD cases in China, 8 cases (8/20) had a mutation at p.R457H, 3 cases (3/7) had a homozygous mutation, and 4 cases (4/7) were compound heterozygote for this mutation, with exlusion of 1 case with missing data. This suggests that p.R457H is a hot-spot mutation in the Chinese population, which is similar to the results reported in other countries. The affected child in our case-series was compound heterozygous for mutations in *POR*: the c.1370 G>A (p.R457H) variant from the mother has been reported as a pathogenic variant in multiple PORD cases, while the c.1379 C>A(p.S460Y) variant from the father has not been reported to date. This variant is “likely pathogenic” according to the American College of Medical Genetics guideline for variant classification (PMID: 25741868). Current bioinformatics analysis suggests that it is necessary to investigate the relationship between the novel mutation site and changes in enzyme function and activity further, to confirm that this genetic change leads to changes in enzyme activity.

In terms of treatment, multidisciplinary cooperative management is required for PORD. High-risk populations for PORD should be identified and screened (27). The patient in this study demonstrated virilisation during pregnancy, but the clinician’s lack of awareness of the disease led to a family tragedy. If timely detection and prenatal diagnosis were implemented, adverse outcomes could be avoided. Careful attention should be paid to the following populations: those with a family history of CAH or PORD; those with maternal virilisation during pregnancy; those with hermaphroditism or skeletal deformity after birth; and those with delayed puberty development. Tests for related hormones and metabolites can be performed in these populations. Genetic testing is helpful for early diagnosis and differential diagnosis of PORD. In a large number of asymptomatic patients (homozygous or compound heterozygotes for autosomal recessive inheritance) and PORD gene carriers (heterozygotes for autosomal recessive inheritance), the disease is more likely to be missed. Typically, mothers with PORD foetuses have low serum estriol levels, which may be detected during the triple antenatal screening test. Subsequent maternal urinalysis may reveal characteristic manifestations of aberrant steroid precursors, which can facilitate a prenatal diagnosis (13, 28). It is necessary to inform mothers that, once virilisation occurs during pregnancy, it should be dealt with as soon as possible. Preconception health education and genetic counselling are required for patients with confirmed PORD or in the above-mentioned high-risk populations. Patients with confirmed PORD need individualised guidance for better natal and prenatal care. It is recommended that spouses should undergo genetic testing to screen for heterozygous cases before conception, or genetic diagnosis should be performed before embryo implantation. Prenatal genetic screening or amniotic fluid cell testing under ultrasonic should be performed during pregnancy for early identification of foetuses with disease genes and for managing the corresponding risks. For neonates born with hermaphroditism or characteristic skeletal deformities, chromosomal examinations are required to determine the genetic sex. Genetic testing is helpful in diagnosing PORD and distinguishing it from other types of CAH. Adrenal gland (blood ACTH, cortisol, electrolytes, and acid–base balance) and gonadal function should be evaluated in affected children, and the detection items that cannot be assessed but that have important diagnostic value (such as 17-hydroxyprogesterone) should be tested elsewhere before treatment. Timely diagnosis and treatment can avoid severe dehydration, electrolyte imbalance, and adrenal cortical crisis, and thus reduce mortality. The patient(i.e. the mother) should be informed of the need for long-term follow-up after birth, with re-examination of 17-hydroxyprogesterone in 2 weeks. Continued increase in blood 17-hydroxyprogesterone concentration is an important diagnostic indicator of 21-OHD. For patients diagnosed in puberty, ACTH stimulation test should be used to determine the degree of glucocorticoid deficiency. Glucocorticoids should be supplemented as appropriate, and drugs should be administered to improve and restore the patient’s secondary sexual characteristics in puberty. If necessary, orthopaedic treatment is needed.

In conclusion, PORD is a group of autosomal recessive genetic disorders. Case 1(the mother) presented signs of virilisation during pregnancy in female foetus, and gave born to a male infant with PORD and a female infant with suspected PORD, and did not receive a timely and precise diagnosis. Through this case report and by reviewing other cases that have been reported in China, we hope to help physicians to understand the rare disease and avoid misdiagnosis. Attention should be paid to the existence of PORD during differential diagnosis of CAH. *POR* mutation can result in various clinical manifestations, including pseudohermaphroditism at birth, skeletal deformity, maternal hyperandrogenism during pregnancy, and adrenocortical insufficiency. Currently, the diagnosis is mainly based on clinical manifestations, abnormal secretion of steroid hormones, and genetic testing. For clinically suspected patients in whom confirmation by biochemical diagnosis is difficult, genetic analysis is recommended. Among the known mutations, POR p.R457H is a hot-spot mutation in the Chinese population. The variant c.1379 C>A (p.S460Y), identified in this study, is a novel mutation in the Chinese population, which enriches the mutation spectrum in *POR* in the Chinese population. The clinical management of PORD requires multidisciplinary cooperation. Prenatal diagnosis, based on the genotypes of probands and their parents, should be provided for families in which subsequent pregnancies are expected.

## Data availability statement

The original contributions presented in the study are included in the article/supplementary material. Further inquiries can be directed to the corresponding authors.

## Author contributions

Manuscript writing, contributed to conception and design of study: JZ. Literature review and sections of manuscript: KW, YoH. Statistical analysis and organizing database: YiH, YiL. Experimental support: SWu, XP. Contributed to conception and design of study: YaL, LY. All authors contributed to manuscript revision, read, and approved the submitted version.

## Funding

This study was supported by the GuangDong Clinical Research Center for Metabolic Diseases (2020B1111170009).

## Acknowledgments

We would like to thank the participation of the patients with PORD. We wish to acknowledge the other members of the Department of Endocrinology for excellent technical assistance, valuable suggestions and/or critical comments.

## Conflict of interest

The authors declare that the research was conducted in the absence of any commercial or financial relationships that could be construed as a potential conflict of interest.

## Publisher’s note

All claims expressed in this article are solely those of the authors and do not necessarily represent those of their affiliated organizations, or those of the publisher, the editors and the reviewers. Any product that may be evaluated in this article, or claim that may be made by its manufacturer, is not guaranteed or endorsed by the publisher.
